# Two Pathways for the Activity-Dependent Growth and Differentiation of Synaptic Boutons in *Drosophila*

**DOI:** 10.1523/ENEURO.0060-19.2019

**Published:** 2019-08-22

**Authors:** Alexander Vasin, Nadezhda Sabeva, Carol Torres, Sébastien Phan, Eric A. Bushong, Mark H. Ellisman, Maria Bykhovskaia

**Affiliations:** 1 Neurology Department, Wayne State University, Detroit, Michigan 48201; 2 Neuroscience Department, Universidad Central Del Caribe, Bayamon, 00957, Puerto Rico; 3 National Center for Microscopy and Imaging Research, University of California San Diego, La Jolla, California 92093

**Keywords:** *Drosophila*, electron tomography, neuromuscular, synapse formation, synapsin, synaptic vesicle

## Abstract

Synapse formation can be promoted by intense activity. At the *Drosophila* larval neuromuscular junction (NMJ), new synaptic boutons can grow acutely in response to patterned stimulation. We combined confocal imaging with electron microscopy and tomography to investigate the initial stages of growth and differentiation of new presynaptic boutons at the *Drosophila* NMJ. We found that the new boutons can form rapidly in intact larva in response to intense crawling activity, and we observed two different patterns of bouton formation and maturation. The first pathway involves the growth of filopodia followed by a formation of boutons that are initially devoid of synaptic vesicles (SVs) but filled with filamentous matrix. The second pathway involves rapid budding of synaptic boutons packed with SVs, and these more mature boutons are sometimes capable of exocytosis/endocytosis. We demonstrated that intense activity predominantly promotes the second pathway, i.e., budding of more mature boutons filled with SVs. We also showed that this pathway depends on synapsin (Syn), a neuronal protein which reversibly associates with SVs and mediates their clustering via a protein kinase A (PKA)-dependent mechanism. Finally, we took advantage of the temperature-sensitive mutant *sei* to demonstrate that seizure activity can promote very rapid budding of new boutons filled with SVs, and this process occurs at scale of minutes. Altogether, these results demonstrate that intense activity acutely and selectively promotes rapid budding of new relatively mature presynaptic boutons filled with SVs, and that this process is regulated via a PKA/Syn-dependent pathway.

## Visual Abstract

**Figure:**
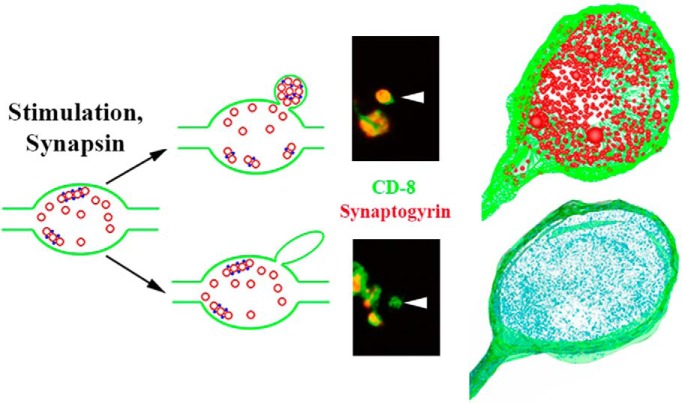

## Significance Statement

Neurons can grow and form new synapses in response to intense activity. We investigated the stages of synapse formation at intact and dissected *Drosophila* larvae and identified a very rapid initial step, which is especially sensitive to nerve stimulation. Specifically, we demonstrated that intense activity triggers budding of new synaptic boutons filled with vesicles, and this pathway becomes very prominent under the conditions of pathologic activity, such as seizures. We found that this pathway depends on protein kinase A and its target synapsin, the protein regulating clustering of synaptic vesicles. These findings suggest a new function for dynamic vesicle clustering in neuronal development and demonstrate that this mechanism can create a positive feedback loop during seizure activity.

## Introduction

Neuronal networks can be modified in response to activity, with synaptic connections being formed or eliminated (Koles and Budnik, 2012). Growth and maturation of new synapses ultimately involves both presynaptic and postsynaptic changes. Although the postsynaptic processes have been extensively studied, and the main stages of the development of postsynaptic specializations have been defined (Bykhovskaia, 2011; Melom and Littleton, 2011; Piccioli and Littleton, 2014), less is known about the stages of presynaptic differentiation and maturation. *Drosophila* glutamatergic neuromuscular junction (NMJ) represents an excellent model system for the study of early stages of growth, differentiation, and maturation of presynaptic boutons (Van Vactor and Sigrist, 2017).

It has been long recognized that activity shapes neuronal growth in *Drosophila* (Shupliakov et al., 2011). The growth and maturation of new synaptic boutons depends on the conversion of neuron, muscle, and glial activity, and it is regulated by multiple molecular mechanisms, primarily including anterograde Wnt-dependent and retrograde BMP-dependent pathways (Bayat et al., 2011; Melom and Littleton, 2011; Koles and Budnik, 2012; Van Vactor and Sigrist, 2017). Importantly, acute stimulation can induce a rapid formation of synaptic boutons in dissected larval preparations (Ataman et al., 2008). This study demonstrated that new filopodia and boutons can be formed rapidly in response to patterned high K^+^ depolarizations, and subsequent studies (Piccioli and Littleton, 2014; Vasin et al., 2014) have shown that the formation of new boutons can occur within one-half hour in preparations with severed axons, suggesting the importance of mechanisms local to synaptic terminals. The new boutons initially lack postsynaptic specializations, and were therefore termed “ghost boutons”. Ghost boutons were sometimes observed in intact undissected larvae (Ataman et al., 2008), and it was shown that they could either stabilize and develop into mature boutons or become eliminated as a result of glial and muscle activity (Fuentes-Medel et al., 2009).

The studies outlined in the previous paragraph suggest that intense activity *in vivo* promotes rapid growth of ghost boutons followed by their subsequent differentiation and maturation; however, this was not yet demonstrated directly. Furthermore, the presynaptic mechanisms controlling the rapid formation of the ghost boutons and their maturation are not yet completely understood. Recent studies demonstrated that the activity-dependent growth of boutons is associated with the dynamics of actin (Piccioli and Littleton, 2014) and synapsin (Syn; Vasin et al., 2014), the protein which binds to actin and clusters synaptic vesicles (SVs; Bykhovskaia, 2011; Shupliakov et al., 2011).

We combined optical and electron microscopy (EM) to investigate the initial stages of the formation of ghost boutons and filopodia in intact and dissected larvae. We found that intense activity *in vivo*, such as locomotion or seizures, induces rapid budding of new boutons filled with SVs, and that this process depends on Syn phosphorylation by protein kinase A (PKA). We also describe another pathway, which is less sensitive to the stimulation and involves growth of filopodia followed by a formation of less mature boutons devoid of synaptic vesicles. Our results suggest that intense activity induces a Syn-dependent formation of vesicle clusters that bud into new synaptic boutons.

## Materials and Methods

### Drosophila stocks and genetics

Flies were cultured on standard medium at room temperature ∼22°C. Actively crawling third instar larvae of both sexes were used for all the experiments. The GAL4/UAS system was used to drive neuronal expression of all the transgenes (Brand and Perrimon, 1993). The stock expressing mCherry-tagged Synaptogyrin (SG-mCherry) was kindly provided by Dr. J. T. Littleton (Stevens et al., 2012). Syn null mutant (cantonized *syn^97^*) was kindly provided by Dr. Erich Buchner (Godenschwege et al., 2004). The *elav^c155^-GAL4*, *UAS-CD8-GFP*, and *sei^ts2^*stocks were obtained from Bloomington Stock Center.

### Intact larvae preparations

Imaging of intact larvae through cuticulum was performed as described by Andlauer and Sigrist (2012a,b). Larvae were anesthetized with CO_2_. Anesthetized larvae were placed on a glass slide between strips of adhesive tape glued to the glass slide, which served as spacers to prevent extreme compression of the larvae. Larvae were positioned ventral side up and covered with a thin film of VECTASHIELD (Vector Laboratories). NMJs on muscle 28 in hemi-segments 2, 3 4, and 5 were imaged.

Subsequently, the larvae were placed in a Petri dish and locomotion activity was induced by either adding high K^+^ (90 mm KCl) or high Na^+^ (500 mm NaCl) solutions for 3 h. Alternatively, the Petri dish with larvae was placed at 35° C for 25 min and then allowed to stay at room temperature for 5 min; the latter protocol was repeated six times, resulting in 3 h of elevated temperature stimulation. All three paradigms (high K^+^, high Na^+^, and elevated temperature) resulted in continuous crawling activity during the 3 h. Subsequently, the larvae were again anesthetized and imaged.

For the evaluation of crawling activity, the larvae were videotaped. The video recordings were analyzed using ImageJ plugin AnimalTracker (Gulyás et al., 2016).

### Dissected larvae preparations

Third instar larvae were dissected in low-Ca^2+^ hemolymph-like HL3.1 saline (in mm: 70 NaCl, 5 KCl, 20 MgCl_2_, 0.2 CaCl_2_, 10 NaHCO_3_, 5 trehalose, 115 sucrose, 2.5 HEPES-HCl, 2.5 HEPES-NaOH, pH 7.2–7.4) at room temperature. Motor nerves were carefully cut below the ventral nerve cord, and the CNS was removed. The preparation was washed several times with the same low-Ca^2+^ HL 3.1 saline and allowed to rest for 5 min. Muscles 6/7 from abdominal segments 2–4 were imaged. The preparations were stimulated with high-K^+^ saline (in mm: 40 NaCl, 90 KCl, 20 MgCl_2_, 1.5 CaCl_2_, 10 NaHCO_3_, 5 trehalose, 115 sucrose, 2.5 HEPES-HCl, 2.5 HEPES-NaOH, pH 7.2–7.4).

### Confocal imaging

Larval preparations were imaged using a real-time laser-based confocal unit (PerkinElmer Life Science) equipped with a CCD camera (Hamamatsu ORCA ER) using a 60×/1 numerical aperture water-immersion objective (Olympus). *Z*-stacks were taken at a 1 μm step to image the entire NMJ. All images were analyzed using Volocity software (Improvision) and contrasted with identical settings.

### Immunohistochemistry

Larvae were fixed for 45 min in HL 3.1 saline containing 4% formaldehyde. Following washing in PBST (0.1% Triton X-100 containing 1× PBS solution), larvae were pre-incubated in the blocking solution containing 2% normal goat serum, 2% bovine serum albumin, and 0.05% sodium azide for 1 h. Primary antibody was applied overnight at 4°C. The secondary antibody was applied for 4–6 h at room temperature. Antibodies were diluted as follows: mouse anti-DLG (Discs large; 1:100; DSHB); rabbit anti-GluRIII [Glutamate receptor subunit III, 1:100; generous gift from Dr. J. T. Littleton (Blunk et al., 2014)] horseradish peroxidase (HRP) conjugated to AlexaFluor 488 (anti-HRP, 1:200; Jackson ImmunoResearch); Texas Red-conjugated goat anti-mouse (1:200; Santa Cruz Biotechnology); AlexaFluor 568 goat anti-rabbit (1:100; Invitrogen). Confocal imaging of fixed tissue was performed using an oil-immersion 50×/0.9 objective (Olympus).

### FM1-43 loading

The activity-dependent endocytic marker FM1-43 (Invitrogen; 10 μm) was added to the high-K^+^ (90 mm) solution and loaded for 2 min. Then the dye was washed for 5 min in a Ca^2+^-free saline (in mm: 70 NaCl, 5 KCl, 20 MgCl_2_, 0 CaCl_2_, 10 NaHCO_3_, 5 trehalose, 115 sucrose, 2.5 HEPES-HCl, 2.5 HEPES-NaOH, pH 7.2–7.4). Destaining was performed during a 5 min high-K^+^ application with no dye added. FM1-43 fluorescence was quantified as described by Akbergenova and Bykhovskaia (2007). Briefly, confocal stacks were acquired to image the entire bouton, the fluorescence intensity was integrated over the 3D volume of the bouton and divided by the voxel counts, and the background was determined in each field-of-view and subtracted.

### Electron microscopy and tomography

The samples were prepared as described by Sabeva and Bykhovskaia (2017). Briefly, samples were fixed in 4% PFA, 2.5% glutaraldehyde, and 0.2 mm CaCl2 in 0.9 mm cacodylate buffer, pH 7.4, in a microwave oven (Biowave, Ted Pella) at 250 W, 28°C–30°C for 2 min, and additionally kept for 15 min in the same fixative at a room temperature. Then, specimens were postfixed for 1 h in 1% osmium tetroxide and 2% uranyl acetate for 30 min. The samples were dehydrated in a graded series of acetone and water mixtures (50, 75, 95, and 100% acetone). Further, the preparations were embedded in Embed 812 epoxy resin overnight at 60°C (Electron Microscopy Sciences). Preparations were serially sectioned (50 nm thick) using a Leica Ultracut ultra-microtome and visualized using a JEOL 100 CX electron microscope equipped with a Hamamatsu digital camera and AMT software.

For electron tomography, embedded preparations were serially sectioned (250 nm thick) and collected on 1 × 2 mm slot grids with 50 nm thick film (LUXELFilm TEM, LUXEL). These sections were post-stained in 2% aqueous uranyl acetate solution for 20 min followed by air dry for 10 min. The grids were additionally contrasted in Sato’s lead solution for 2–3 min and washed in water. The grids were glow discharged and then immersed in 0.05% BSA/15 nm diameter colloidal gold particles solution to serve as fiducial markers. Grids were carbon coated for additional stability. Single-tilt series were recorded with a JEOL 4000EX electron microscope operated at 400 kV. Serial EM (Mastronarde, 2005) images were collected using an 8 × 8 k CCD camera assembly at 12,000 magnification. Angular increments of 1° (usually from −55° to +55°) about an axis perpendicular to the optical axis of the microscope were achieved using a computer-controlled goniometer. The pixel resolution was 0.625 nm. TxBR was used to align and reconstruct the 3D tomograms (Lawrence et al., 2006). The IMOD (Kremer et al., 1996) software package was used for data segmentation.

### Statistical analysis

Multiple comparisons were performed using one-way or two-way ANOVA followed by Tukey test, whereas paired comparisons were performed using two-sided unpaired *t* test.

## Results

To investigate the initial stages of the activity-dependent formation and differentiation of new boutons *in vivo*, we imaged NMJs in intact larvae on intense crawling activity. The NMJ at the ventral muscle 28 was imaged through cuticulum in anesthetized larvae expressing the neuronal marker CD8-GFP. Then the larva was placed in a Petri dish, and crawling activity was induced for 3 h. Subsequently, the larva was again anaesthetized and imaged. In control preparations, larvae were placed in a Petri dish for 3 h but not stimulated.

**Figure 1.**
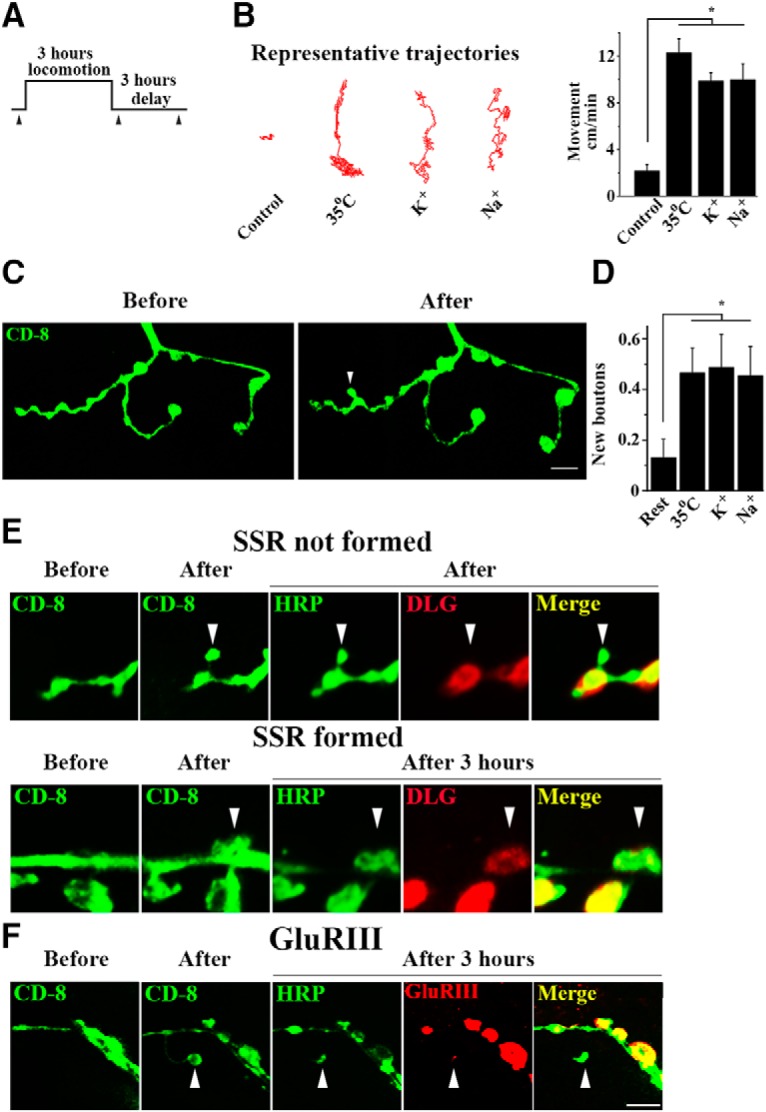
Locomotion activity acutely induces robust outgrowth of new presynaptic boutons, which subsequently acquire postsynaptic specializations. ***A***, The diagram illustrating the experimental protocol. The arrowheads point to the imaging time points. ***B***, Locomotion activity is significantly promoted by either treatment: elevated temperature or high K^+^ or high Na^+^ solutions. Representative trajectories of moving larva are shown on the left. A larvae position in a trajectory is represented by a single pixel. The movement distance was obtained as a sum of all the pixels. Asterisk indicates *p* < 0.0001 per 1-way ANOVA. ***C***, The NMJ (CD8-GFP fluorescence) imaged in intact larvae at muscle 28 before and after the 3 h period of locomotion activity. The arrowhead points to a new bouton. Scale bar, 5 µm. ***D***, The number of new boutons is significantly increased by locomotion activity (Asterisk indicates *p* < 0.05, per 1-way ANOVA). The data collected from at least 26 NMJs (4 larvae) per condition. ***E***, Immunostaining for neuronal (HRP, green) and postsynaptic SSR (DLG, red) markers show that after stimulation the postsynaptic DLG marker is absent (top). However, in the preparations fixed after the 3 h delay the DLG marker is often present (bottom). The arrowhead points to a new bouton. ***F***, Postsynaptic glutamate receptor GluRIII (red) is often present in the preparations fixed after the 3 h delay following the stimulation. Scale bar, 5 µm.

Crawling activity was induced by three different methods. First, we built on an earlier study, which demonstrated that elevated temperatures promote locomotion and synaptic growth (Sigrist et al., 2003). Locomotion was induced for the duration of 3 h (Fig. 1*A*) by repeatedly placing the larvae at 35° C for 25 min and then allowing it to stay at room temperature for another 5 min; this procedure was repeated six times. This paradigm produced continuous crawling activity (Fig. 1*B*), as well as robust synaptic growth of filopodia and ghost boutons (Fig. 1*C*). To ensure that the observed effect is induced by locomotion and not by some nonspecific effects of elevated temperatures, we also induced continuous crawling activity by placing the larvae in a dish covered by a thin layer of saline (either 500 mm NaCl or 90 mm KCl) for 3 h (Fig. 1*B*). All the paradigms produced similar synaptic growth (Fig. 1*D*).

To investigate whether the new boutons form postsynaptic specializations, we first performed immunostaining for the postsynaptic marker DLG (Lahey et al., 1994), which labels the subsynaptic reticulum (SSR). The larvae were fixed either immediately after the locomotion paradigm and live imaging, or after a delay of additional 3 h. Immunostaining for the neuronal marker HRP (Jan and Jan, 1982) was also performed to clearly label presynaptic boutons in fixed preparation. We found that in the preparations fixed immediately after the locomotion new boutons were always lacking postsynaptic specializations (Fig. 1*E*, top). In contrast, the preparations fixed after a 3 h delay had sometimes (but not always) a formed SSR (Fig. 1*E*, bottom). To investigate whether the new postsynaptic specializations become functional, we performed immunostaining for postsynaptic receptors GluRIII (Marrus et al., 2004; Blunk et al., 2014). We found that in the preparations fixed with a 3 h delay following the activity some of the new boutons showed clear GluRIII immunoreactivity (Fig. 1*F*). These results suggest that functional postsynaptic specializations are formed following the outgrowth of new presynaptic boutons within hours.

**Figure 2.**
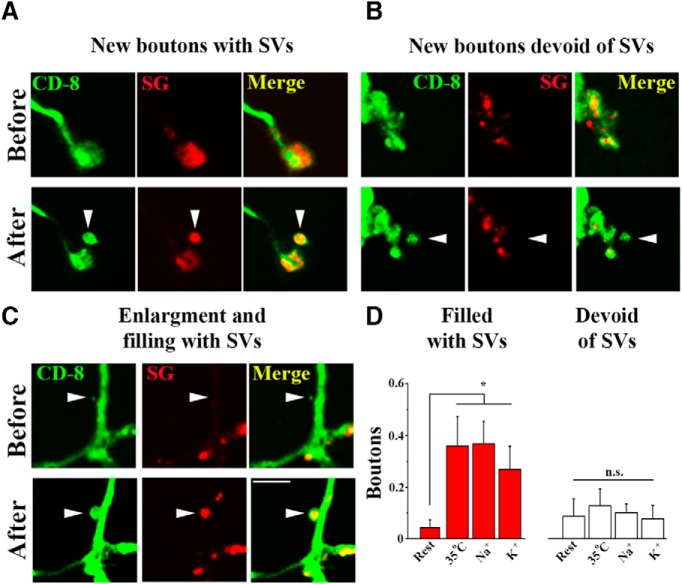
Locomotion activity selectively promotes the growth of synaptic boutons filled with SVs. ***A***–***C***, SV marker SG-mCherry indicates that the population of new boutons is highly heterogeneous. New boutons sometimes possess (***A***) and sometimes lack (***B***) the SG-mCherry marker, and some of the boutons acquire the SG marker on locomotion (***C***). Scale bar, 5 µm. ***D***, The number of new SV-filled boutons is significantly increased by locomotion activity (red bars; Asterisk indicates *p* < 0.05, per 1-way ANOVA), whereas the number of SV-devoid boutons is not (white bars; not significant, *p* > 0.05, per 1-way ANOVA). The data collected from at least 26 NMJs (4 larvae) per condition.

Next, we investigated how the new boutons acquire SVs. To do this, we took advantage of the line expressing the SV marker SG-mCherry and combined in with the neuronal membrane marker CD8-GFP. Interestingly, we observed considerable heterogeneity of the SG-mCherry marker within the population of newly formed boutons (Fig. 2). The majority of new boutons had bright mCherry labeling (Fig. 2*A*), suggesting that these boutons were filled with SVs. However, some new boutons, as well as filopodia, were totally lacking the SG-mCherry marker (Fig. 2*B*). Interestingly, we also observed boutons initially lacking the SG-mCherry marker, which acquired it following the locomotion activity (Fig. 2*C*), suggesting that theses boutons were initially devoid of SVs but acquired them following activity. Surprisingly, we found that locomotion activity affected only the numbers of new boutons filled with SVs, whereas the outgrowth of filopodia and boutons devoid of SVs was not affected by activity (Fig. 2*D*).

**Figure 3.**
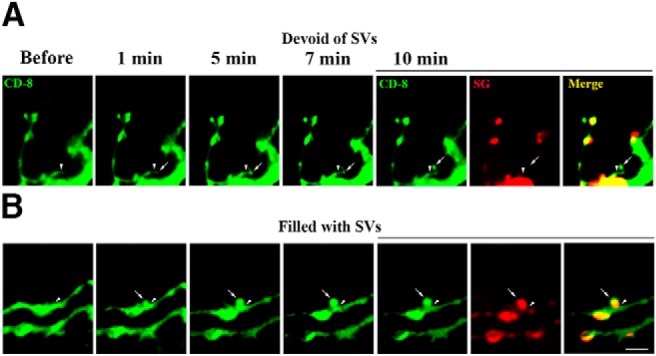
Two patterns of the outgrowth of new boutons. Top, Growth of filopodia (1–5 min) resulting in a formation of a new bouton (7–10 min). The new bouton does not have the SG-mCherry marker. Bottom, A new bouton budding from a terminal brunch (1 min) and then growing (5 min) and separating from the brunch (7 min). The bouton has bright and evenly distributed SG-mCherry marker. Arrowheads mark the origins of the growth; arrows mark growing filopodia or new boutons. Scale bar, 5 µm.

These findings can be explained by two different models. First, the boutons devoid of SVs and those filled with SVs could be produced via separate pathways. Alternatively, the boutons devoid of SVs and those filled with SVs represent different stages of synapse formation and maturation. To discriminate between these possibilities, we performed continuous imaging of dissected larvae (abdominal muscles 6 and 7). To promote outgrowth, we imaged the preparations at elevated K^+^ concentration (90 mm KCl). We observed two different patterns of synaptic growth (Fig. 3): a growth of filopodia followed by a formation of a bouton devoid of SVs (Fig. 3*A*), and a rapid budding of a new bouton filled with SVs (Fig. 3*B*). This result supports the first scenario, whereby the boutons initially devoid of SVs and those filled with SVs are formed via different pathways.

**Figure 4.**
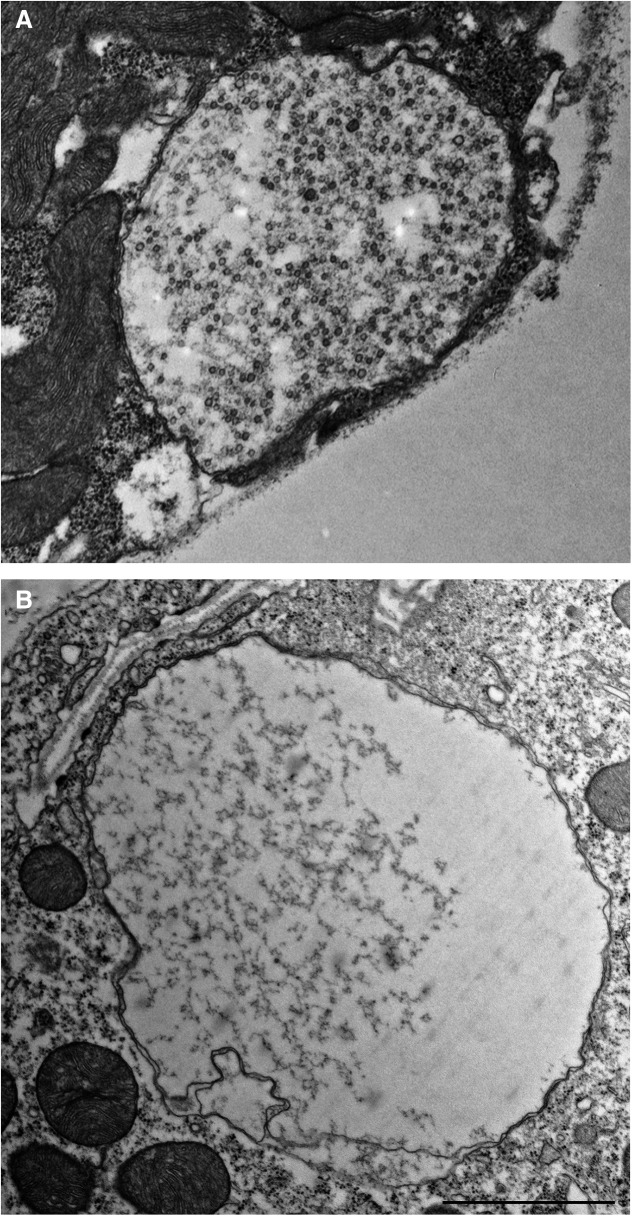
EM analysis reveals two types of ghost boutons: those filed with SVs and those devoid of SVs. ***A***, A ghost bouton is packed with evenly distributed SVs. ***B***, A ghost bouton lacks SVs but contains filamentous matrix. Note a lack of SSR in the muscles surrounding both boutons. Scale bar, 1 µm.

To investigate these two types of new boutons at the ultrastructural level, we performed EM analysis of stimulated NMJs. NMJs were stimulated by three temporally-spaced high K^+^ depolarizations (2 min at 90 mm KCl followed by 10 min rest; Vasin et al., 2014) and processed for EM immediately following the stimulation. Ghost boutons were identified as described by Vasin et al. (2014). We first identified new boutons in dissected CD8-GFP larvae using confocal microscopy, and then applied correlative light/EM analysis. To confirm that the new boutons were identified in electron micrographs correctly, we used the lack of SSR around the boutons as a hallmark for ghost boutons. We found that the majority of ghost boutons were tightly packed with SVs (Fig. 4*A*). However, boutons totally devoid of SVs but containing filamentous matrix were also observed (Fig. 4*B*).

**Figure 5.**
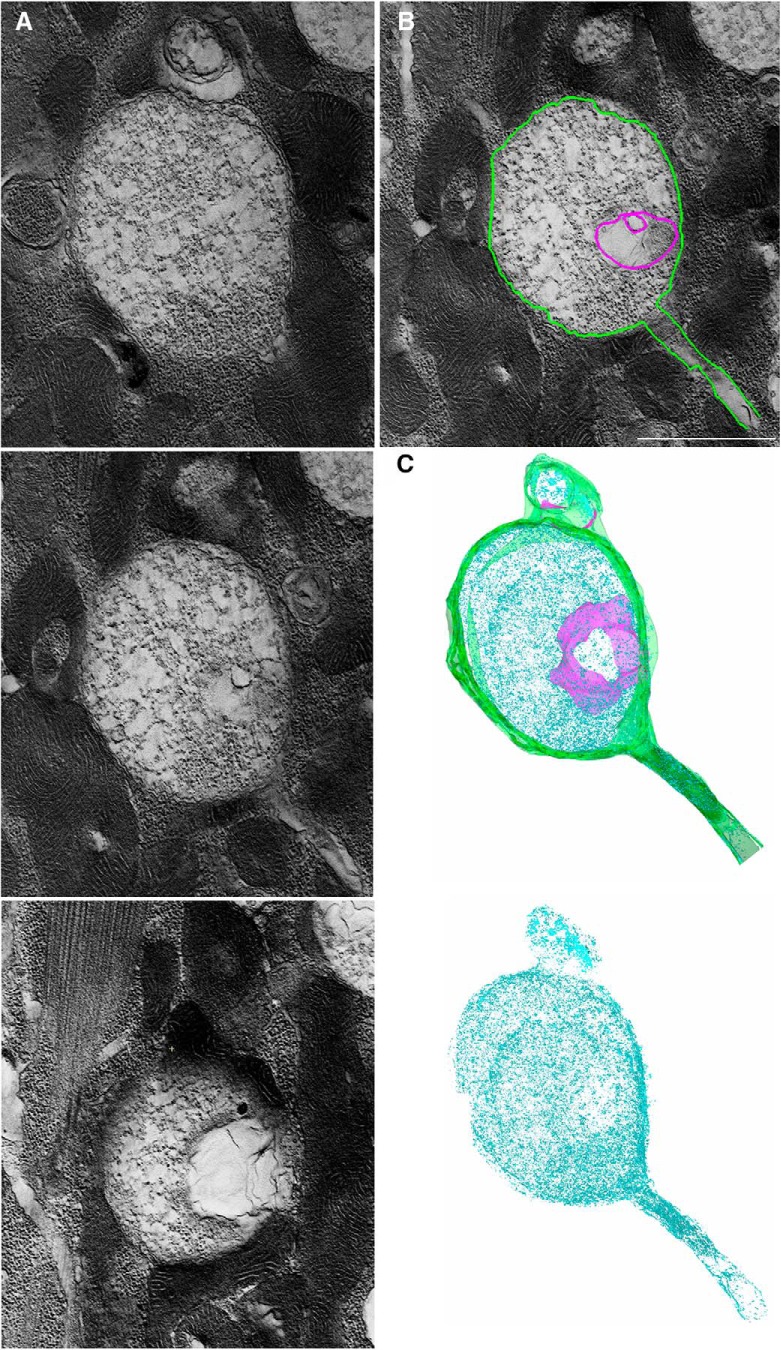
EM tomographic analysis of a ghost bouton devoid of SVs reveals densely packed filaments, membranous folds, and a connected filopodia. ***A***, Three computed slices taken from different positions within tomogram of a ghost bouton. ***B***, External (green) and internal (purple) membranes are outlined in a tomogram. Scale bar, 1 µm. ***C***, The reconstructed volume (1.5 µm thickness, 6 serial sections) showing filamentous matrix (cyan), internal membranous folds (purple), and the external membrane (green). Bottom, Membranes are excluded to show filaments densely packed throughout the reconstructed volume.

**Figure 6.**
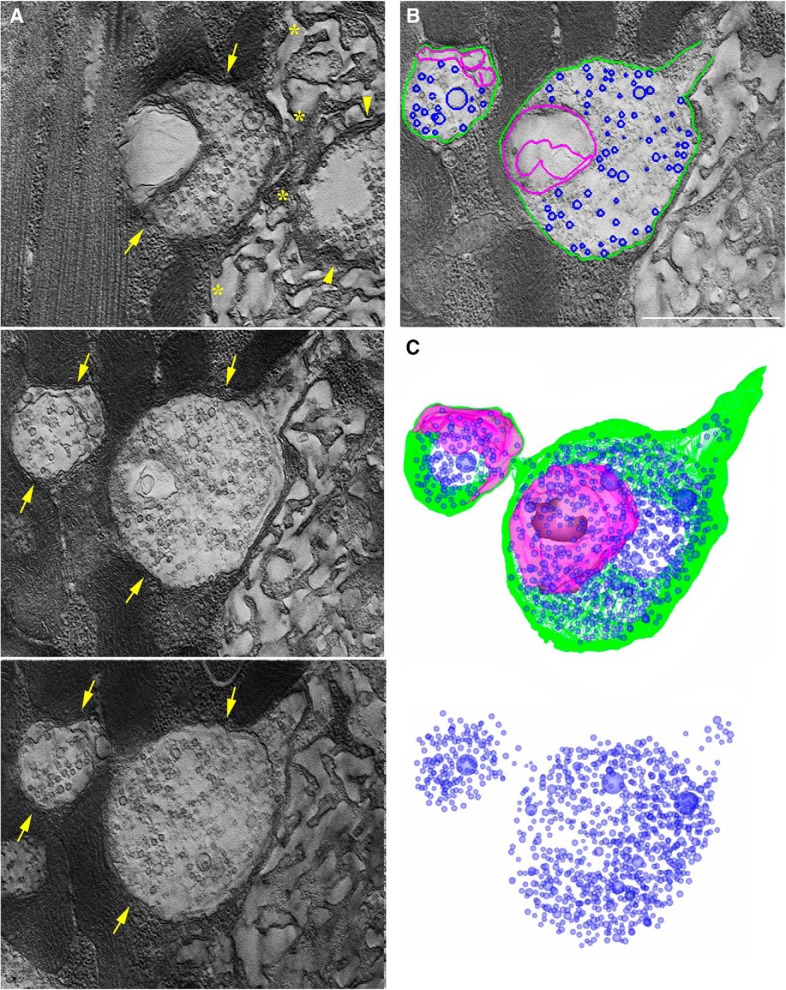
EM tomographic analysis of ghost boutons filled with SVs. ***A***, Three computed slices showing two interconnected ghost boutons (arrows) at the edge of the SSR (marked by *) surrounding a mature bouton (top plane, arrowheads). ***B***, External (green) and internal (purple) membranes as well as SVs (blue) are outlined. Scale bar, 1 µm. ***C***, The reconstructed volume (1.5 µm thickness) showing both ghost boutons, which are interconnected, uniformly filled with SVs (blue), and contain membranous folds (purple).

To perform a more detailed characterization of the ultrastructure of ghost boutons, we used EM tomography coupled with serial sectioning and 3D reconstructions. We performed tomography analysis for two different preparations and were able to identify and partially reconstruct five boutons packed with SVs and four boutons devoid of SVs. We found that the latter boutons were packed with filamentous matrix and sometimes contained internal membranous folds (Fig. 5). The reconstructed volumes of these boutons were totally lacking SVs. In contrast, the boutons of the other type were tightly packed with SVs, and they also sometimes contained membranous folds (Fig. 6). Overall, our EM analysis revealed two different types of ghost boutons: those less mature, lacking SVs, and filled with filamentous matrix and membrane folds (Fig. 4), and those more mature, packed with SVs, and possibly capable of exocytosis/endocytosis (Fig. 5).

**Figure 7.**
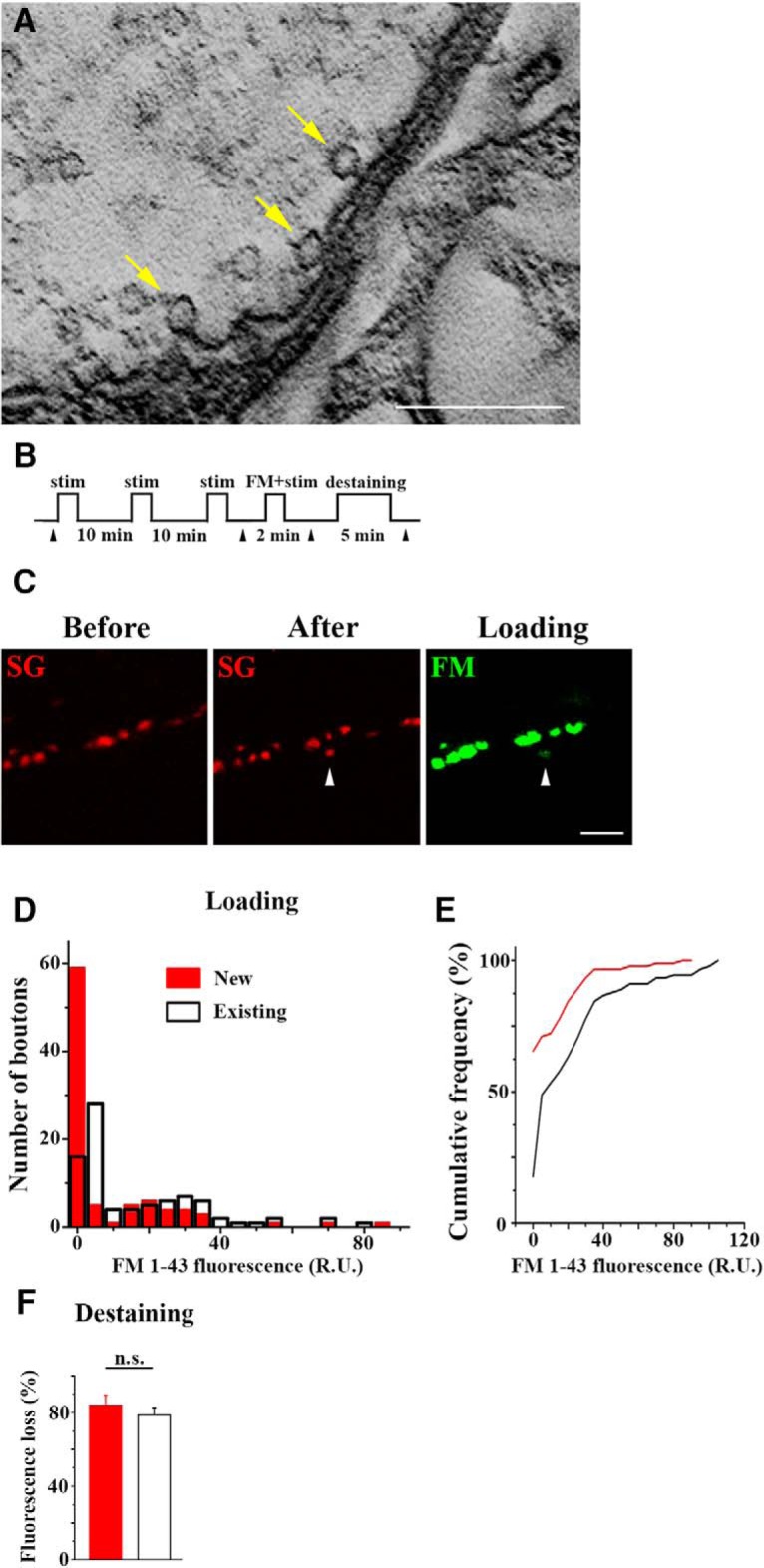
New boutons filled with SVs are capable of exocytosis/endocytosis. ***A***, SVs docked (arrows) and partially fused (arrowhead) with the plasma membrane. Scale bar, 0.5 µm. ***B***, A diagram showing the experimental protocol: three temporally-spaced high K^+^ stimulations are followed by 2 min FM1-43 loading and 5 min destaining. Arrowheads show the imaging time-points. ***C***, A new SV-filled bouton (arrowhead) is formed after the patterned depolarization has acquired the endocytic marker FM1-43. ***D***, The distribution of FM1-43 fluorescence in new and mature boutons shows that the fraction of new boutons (red bars) has uptaken FM1-43 dye, and that the range of FM1-43 fluorescence in new boutons is comparable to that in mature boutons (white bars). The datasets includes 90 new and 90 mature boutons. ***E***, The cumulative distribution of FM1-43 fluorescence shows that the overall FM1-43 uptake in new boutons is significantly smaller (*p* < 0.0001 per K–S test) than that in mature boutons. ***F***, Destaining levels were similar in new and mature boutons (the new boutons that did not acquire FM1-43 were excluded from the analysis). n.s. = not significant.

Interestingly, in more mature boutons packed with SVs we sometimes observed SVs docked to the plasma membrane and partially fused (Fig. 7*A*). These observations pointed to possible exocytic/endocytic processes in new boutons. To test whether this is the case, we used the activity-dependent lipophilic dye FM1-43, which becomes uptaken by SVs during endocytosis. These experiments were performed with the line expressing only SG-mCherry (but not CD8-GFP, so that only FM1-43 dye would be detected by the green channel). The experiments were performed at dissected preparations as illustrated in Figure 7*B*. Patterned depolarization (3 high K^+^ applications) was used to produce robust outgrowth (Ataman et al., 2008; Piccioli and Littleton, 2014; Vasin et al., 2014). This protocol enabled us to detect only the new boutons containing SVs, because the neuronal marker was absent. Subsequently, FM1-43 dye was loaded to identify the boutons involved in endocytic processes. Finally, the preparations were destained using high K^+^ stimulation with no dye added to test whether exocytosis takes place.

We identified 90 new boutons filled with SVs in 11 preparations and found that >30% of the new boutons acquired FM1-43 dye (Fig. 7*C*–*E*). As a baseline, we also quantified FM1-43 loading of the existing mature boutons. To eliminate the selection bias, for each new bouton we selected an adjacent mature bouton from the same field-of-view. As illustrated in Figure 7, *C* and *D*, FM1-43 loading in new boutons was not as prominent as in mature boutons, and the majority of new boutons did not acquire detectable levels of FM1-43. However, >30% of new boutons demonstrated prominent FM1-43 loading, which was comparable to that in mature boutons. Furthermore, all the boutons loaded with FM1-43, including new and mature ones, distained by ∼80% during a subsequent stimulation with no dye added (Fig. 7*F*; the boutons that did not acquire FM1-43 were excluded from the distaining analysis). These results combined with the EM analysis (Fig. 7*A*) demonstrate that the new boutons filled with SVs are capable of SV fusion and recycling.

**Figure 8.**
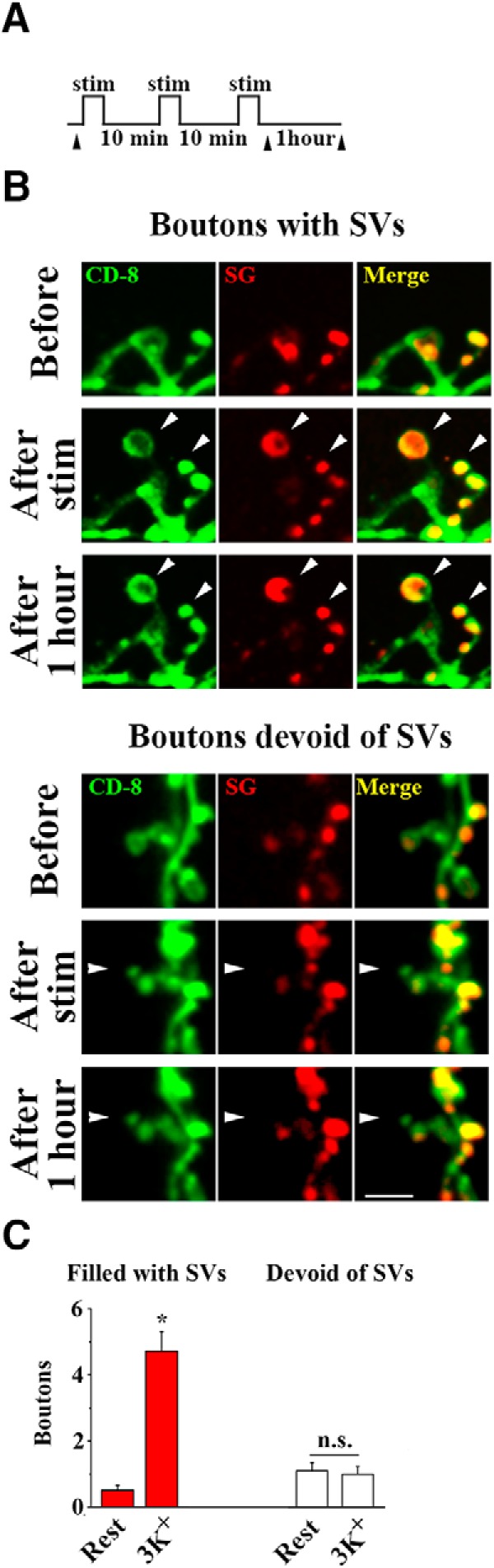
Patterned depolarization selectively promotes the growth of SV-filled boutons. ***A***, A diagram showing the experimental protocol: three temporally-spaced high K^+^ stimulations (2 min each) are followed by a 1 h delay. Arrowheads show the imaging time-points. ***B***, Boutons filled with SVs labeled with SG-mCherry (top) or devoid of SVs (bottom) appear after the high K^+^ stimulation and remain intact and unchanged after a 1 h delay. Scale bar, 5 µm. ***C***, The numbers of new SV-filled boutons are significantly enhanced by high K^+^ stimulation (red bars; asterisk indicates *p* < 0.001, per 1-way ANOVA), whereas the numbers of new boutons devoid of SV are not different for stimulated and resting (for 26 min) preparations (white bars; *p* > 0.05 per1-way ANOVA). Data collected from 33 NMJs (12 larvae) for each condition. n.s. = not significant.

Next, we questioned how the new boutons devoid of SVs mature, and whether and when they acquire SVs. To address this question, we used the patterned depolarization at dissected preparations expressing both CD8-GFP and SG-mCherry and examined the preparations immediately after the stimulation and following a 1 h delay (Fig. 8*A*). We observed both types of the new boutons and found that both types remained unchanged during the 1 h delay that followed the stimulation (Fig. 8*B*). The less mature boutons never acquired SVs within the resting period, but they remained stable (Fig. 8*B*, bottom).

Notably, the stimulation affected only the numbers of new boutons filled with SVs (Fig. 8*C*, left). In contrast, the numbers of new boutons devoid of SVs were similar in stimulated and control preparations (Fig. 8*C*, right; control preparations remained at rest for 26 min to match the length of the stimulation protocol). This result agrees with the results obtained in intact larvae (Fig. 2*C*). Altogether, these results suggest that new boutons are formed via two different pathways, and that intense activity promotes only one of the pathways, namely rapid budding of boutons filled with SVs.

**Figure 9.**
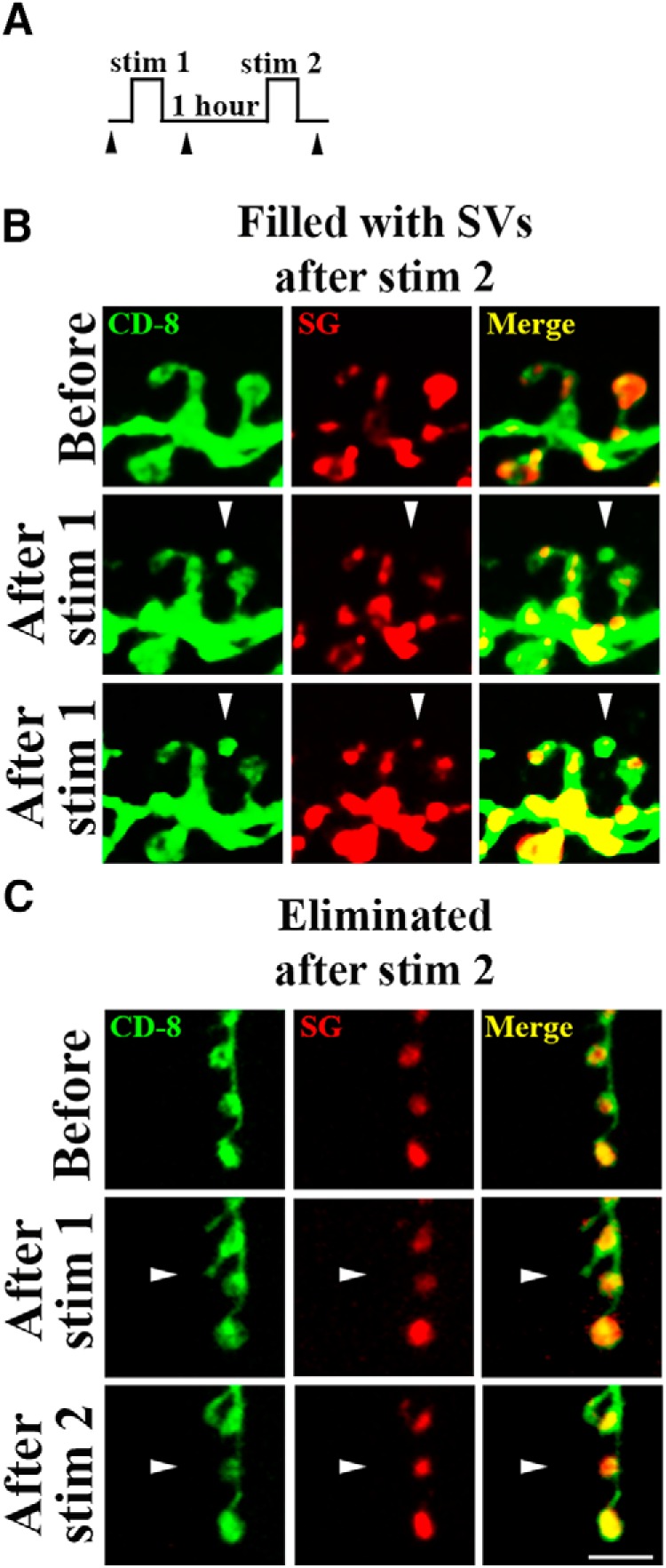
The new boutons devoid of SVs may either acquire SVs or degrade on a subsequent stimulation. ***A***, A diagram showing the experimental protocol: two high K^+^ stimulations (2 min each) are separated by a 1 h interval. Arrowheads show imaging time points. ***B***, A new bouton initially devoid of SVs has acquired the SG-mCherry marker on the second stimulation. ***C***, A new bouton devoid of SVs was eliminated on the second stimulation. Scale bar, 5 µm.

We then questioned whether activity would promote the maturation of new boutons that were initially formed being devoid of SVs. To elucidate this question, we delivered two high K^+^ depolarizations (90 mm for 2 min) in dissected preparations (Fig. 9*A*); the depolarizations were spaced by a 1 h delay, and we imaged NMJs after each depolarization. Although the outgrowth produced by a single depolarization was modest, we were able to detect both types of new boutons. Notably, some of the new boutons initially devoid of SVs did acquire the SG-mCherry marker after a subsequent depolarization (Fig. 9*B*). Furthermore, some of the new boutons devoid of SVs were eliminated after a subsequent stimulation (Fig. 9*C*). In contrast, we did not observe any elimination of the boutons filled with SVs.

**Figure 10.**
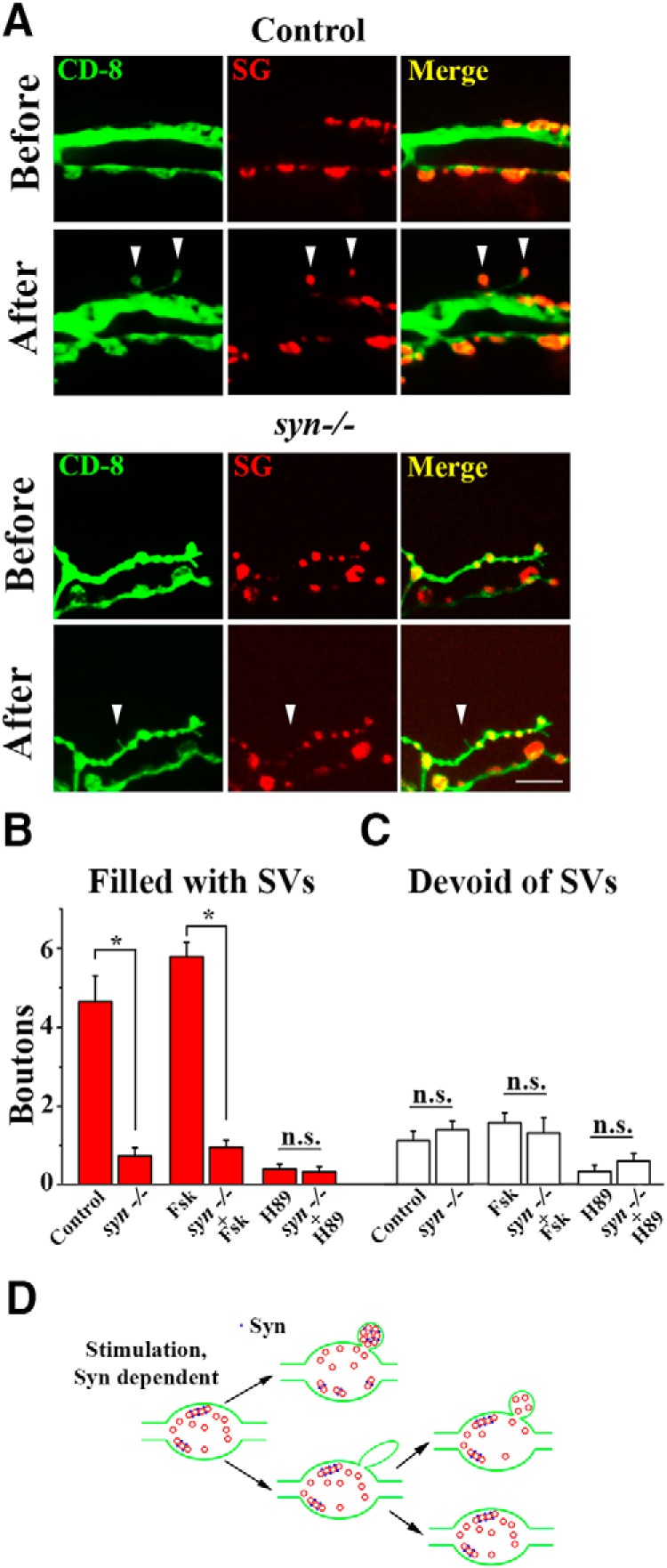
The growth of the boutons filled with SVs depends on the PKA/Syn pathway. ***A***, Outgrowth of SV-filled boutons is not prominent in *syn-/-* preparations. Scale bar, 5 µm. ***B***, The growth of SV-filled boutons is reduced in *syn-/-* preparations. (Asterisks indicate *p* < 0.0001 per 2-way ANOVA). ***C***, The growth of SV-devoid boutons is unaffected by Syn deletion. (n.s. *p* > 0.05 per 2-way ANOVA) Data collected from 27 NMJs (5 larvae) for each genotype at each condition; the outgrowth is significantly affected by the treatment in both control and *syn-/-* larvae. ***D***, A model illustrating the observed two pathways for neuronal growth: Syn-dependent budding of new boutons filled with SVs and growth of SV-devoid filopodia, which can either stabilize and mature, or become eliminated.

We next asked, what is the mechanism that promotes the activity-dependent budding of SV-filled boutons. We reasoned that Syn would be a likely candidate, because it clusters SVs (Bykhovskaia, 2011; Shupliakov et al., 2011) and redistributes within neuronal terminals in response to activity (Chi et al., 2001; Orenbuch et al., 2012; Vasin et al., 2014). Furthermore, Syn deficiency inhibits synaptogenesis in several preparations (Valtorta et al., 2011), including *Drosophila* (Vasin et al., 2014). To test whether Syn promotes the activity-dependent budding of SV-filled boutons, we took advantage of Syn deleted (*syn-/-*) (Godenschwege et al., 2004) larvae and brought CD8-GFP and SG-mCherry transgenes onto *syn-/-* background. We found that in *syn-/-* larvae the patterned depolarization at dissected preparations (three 2 min high K^+^ applications spaced by 10 min intervals) produced very low outgrowth of the boutons filled with SVs, compared with controls (Fig. 10*A*,*B*). In contrast, the numbers of boutons devoid of SVs were not affected by Syn deletion (Fig. 10*C*). This result shows that Syn mediates budding of new SV-filled boutons, but not the outgrowth of filopodia.

Because Syn activity depends on its phosphorylated state, and because PKA phosphorylation of Syn promotes synapse formation (Valtorta et al., 2011), we next asked whether the PKA phosphorylation of Syn is the mechanism regulating the activity-dependent budding of SV-filled boutons. To address this question, we either promoted PKA activity by forskolin (Fsk) treatment (10 μm applied for 1 h before the stimulation; Vasin et al., 2014) or inhibited PKA activity by H89 treatment (25 μm applied for 20 min before the stimulation; Cho et al., 2015). Both treatments significantly affected the overall outgrowth in control preparations: the average number of new boutons per segment increased from 5.75 ± 0.48 to 7.35 ± 0.31 (*p* < 0.05) on Fsk treatment and decreased to 0.73 ± 0.14 (*p* < 0.001) on H89 treatment. In Syn(−) preparations, Fsk failed to promote the overall growth (2.25 ± 0.26 in Fsk-treated vs 2.11 ± 0.21 in untreated preparations), although it was significantly reduced by H89 treatment (to 0.93 ± 0.16, *p* < 0.01).

We then analyzed separately the boutons filled with SVs and those devoid of SVs in control and *syn-/-* preparations treated with Fsk and H89 (Fig. 10*B*,*C*). Both types of boutons showed the dependence on the treatment, although the overall effect was stronger for SV-filled boutons (*p* = 5 × 10^−9^ per two-way ANOVA and 14.2-fold overall increase from H89 to Fsk-treated preparations for SV-filled boutons vs *p* = 0.002 and 4.7-fold overall increase for SV-devoid boutons). In contrast, only SV-filled boutons showed a significant defect in growth on Syn deletion (*p* = 5 × 10^−12^ per two-way ANOVA; Fig. 10*B*), whereas the growth of SV-devoid boutons was not sensitive to Syn deletion (*p* = 0.83; Fig. 10*C*).

These results demonstrate that the budding of new SV-filled boutons depends on Syn and is promoted by PKA activity. Our results also show that PKA activity affects synaptic growth more broadly than via the Syn-dependent pathway, because the outgrowth of filopodia and SV-devoid boutons is not affected by Syn deletion but does depend on PKA. Altogether, our results support a model, in which new boutons can be formed either via a Syn-dependent budding or via a Syn-independent filopodia outgrowth (Fig. 10*D*).

Finally, we asked how rapidly the SV-filled boutons can form *in vivo*, and how would this pathway be manifested under the conditions of pathologically enhanced activity, such as seizures. To address this question, we took advantage of the temperature-sensitive seizure mutant *sei* (Jackson et al., 1984, 1985). This line has a mutation in *erg* gene encoding a voltage-activated K^+^ channel (Titus et al., 1997). On elevated temperatures (>37°), *sei* flies show bouts of uncontrolled flight activity followed by paralysis, whereas *sei* larvae at elevated temperatures demonstrate intense motor activity, such as twitching and contractions.

**Figure 11.**
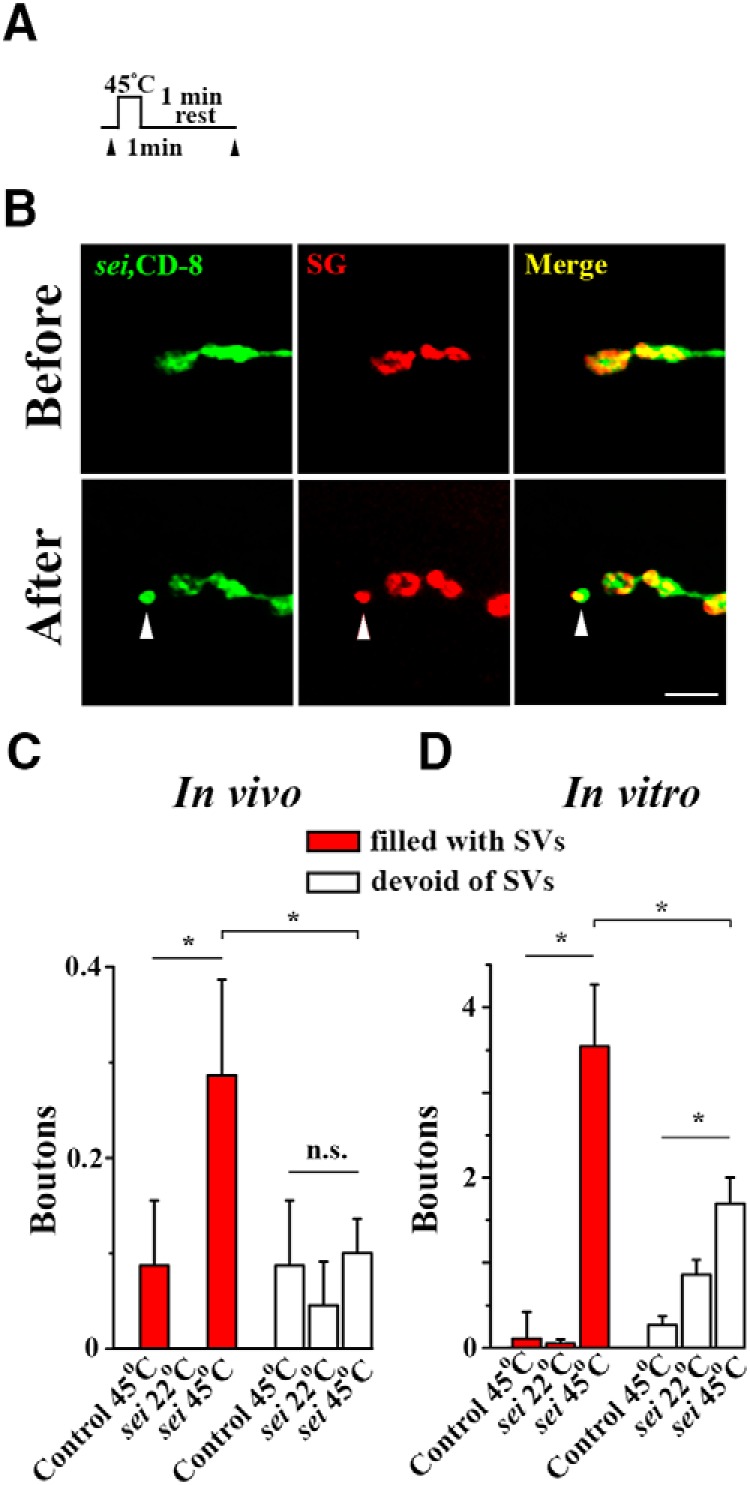
Seizure activity promotes a rapid outgrowth of SV-filled boutons. ***A***, Diagram showing the experimental protocol: intact or dissected larvae were placed at 45°C for 1 min and then allowed to relax for 1 min at a room temperature (∼22°C). ***B***, A new SV-filled bouton in an intact *sei* larva. Scale bar, 5 µm. ***C***, ***D***, Both intact (***C***) and dissected (***D***) *sei* larvae show a significant outgrowth of SV-filled boutons on elevated temperature (Asterisk indicates red bars; *p* < 0.01, per 1-way ANOVA). The outgrowth of SV-filled boutons in *sei* larva at elevated temperatures significantly exceeds the outgrowth of SV-devoid boutons (Asterisk indicates *p* < 0.05, per 1-way ANOVA). n.s. = not significant.

To investigate whether seizure activity acutely affects neuronal growth, we brought CD8-GFP and SG-mCherry transgenes onto *sei* background. The experiments were performed at both intact and dissected larvae. Larvae were placed at 45° for 1 min and then allowed to relax for 1 min at a room temperature (Fig. 11*A*). This protocol produced robust outgrowth of new boutons in both intact (Fig. 11*B*) and dissected larvae. For controls, we used Canton S. larva that underwent a similar temperature exposure, as well as *sei* larvae that were left at a room temperature for 2 min. Both dissected and intact *sei* larvae demonstrated a significant outgrowth of new boutons on elevated temperatures (Fig. 11*C*,*D*). The growth of SV-filled boutons was significantly promoted by seizure activity in both intact and dissected preparations (Fig. 11*C*,*D*, red bars). The growth of SV-devoid boutons and filopodia in dissected preparations (Fig. 11*D*, white bars) was not as prominent as the growth of SV-filled boutons (Fig. 11*D*, red bars), although it was statistically significant compared with controls. No significant growth was detected for SV-devoid boutons and filopodia in intact preparations (Fig. 11*C*, white bars). This result demonstrates that very intense stimulation, such as seizures, can trigger robust budding of new synaptic boutons within minutes.

## Discussion

The main finding of our study is that intense neuronal activity, such as seizures or locomotion, can trigger very rapid budding of synaptic boutons filled with SVs at a time scale of minutes, and this process depends on the activity of PKA and its target Syn. We found that in the absence of intense activity neuronal growth occurs via two different pathways: (1) rapid budding of boutons filled with SVs; and (2) a slower growth of filopodia, which initially produces less mature boutons devoid of SVs and filled with filamentous matrix. The latter subtype can subsequently either mature and acquire SVs, or become eliminated. We found that intense activity, such as continuous crawling or seizures, predominantly promotes the rapid PKA/Syn-dependent budding of more mature boutons. These results suggest that Syn-dependent SV clustering may serve as a trigger for rapid activity-dependent budding of new boutons.

Our findings elucidate the initial steps of synapse formation. The structure and organization of neuronal terminals is very complex, and therefore building new synapses requires coordinated activity of presynaptic, postsynaptic, and glial cells (Freeman, 2006; Griffith and Budnik, 2006; Colon-Ramos, 2009; Corty and Freeman, 2013; Van Vactor and Sigrist, 2017). Synaptic terminals include highly specialized presynaptic and postsynaptic membranes and protein complexes (Sudhof, 2018), SVs organized into several functional pools (Schneggenburger and Rosenmund, 2015; Neher and Brose, 2018), active zones that scaffold protein complexes required for exocytosis (Petzoldt et al., 2016; Chen et al., 2018; Ehmann et al., 2018), and the machinery that mediates multiple components of endocytosis (Shupliakov, 2009; Saheki and De Camilli, 2012; Cousin, 2017; Li and Kavalali, 2017). Respectively, the formation of new synapses requires protein synthesis and active axonal transport (Sudhof, 2018). These processes can be promoted by neuronal activity via anterograde and retrograde signaling, predominantly involving *Wnt*-dependent (Koles and Budnik, 2012; Speese et al., 2012) and BMP-dependent molecular pathways (McCabe et al., 2003; Berke et al., 2013; Piccioli and Littleton, 2014).

The *Drosophila* larval NMJ is an excellent model system broadly used to study synapse formation (Melom and Littleton, 2011; Koles and Budnik, 2012; Van Vactor and Sigrist, 2017). In this preparation, new synaptic boutons can be formed acutely in response to patterned depolarization (Ataman et al., 2008). This important finding opened up opportunities for investigating the initial stages of synapse formation and differentiation. In particular, this study demonstrated that the growth of new synaptic connections starts from the formation of presynaptic boutons, which initially lack postsynaptic specializations. Subsequent studies (Piccioli and Littleton, 2014; Vasin et al., 2014) demonstrated that the formation of new presynaptic boutons can be triggered in preparations with severed axons, suggesting that it depends on the mechanisms local to synaptic terminals.

Here, we focused on the initial stages of the growth and differentiation of presynaptic boutons. We identified a very rapid step in presynaptic formation, which is especially sensitive to acute stimulation. We found that intense activity *in vivo* triggers budding of new boutons filled with SVs and demonstrated that this pathway can become very prominent under the conditions of pathologically intense activity, such as seizures. As such, this mechanism would likely contribute to a positive feedback loop, increasing the number of synaptic connections in response to hyperactivity and further promoting the pathologic condition.

We found that this rapid budding of new boutons filled with SVs is distinct from a slower growth of filopodia, which results in the formation of less mature boutons. We investigated the rapidly formed boutons at the ultrastructural level and found that they are packed with SVs, in contrast to the other type of boutons, which are packed with a filamentous matrix. Our EM tomography analysis combined with live imaging of the activity-dependent endocytic marker FM1-43 demonstrated that some of the rapidly formed new boutons are capable of exocytic/endocytic processes, although they do not possess postsynaptic specializations.

We demonstrated that the rapid budding of the relatively mature SV-packed boutons depends on the SV-associated protein Syn. It has long been recognized that Syn promotes neuronal growth and synapse formation in a PKA-dependent manner (Ferreira et al., 1995; Kao et al., 2002; Valtorta et al., 2011). However, the specific mechanism of this Syn action are not yet fully understood. Interestingly, the activity-dependent growth of new boutons in *Drosophila* tends to occur in the spots of Syn accumulation (Vasin et al., 2014).

It is established that Syn mediates the formation of dynamic SV clusters (Shupliakov et al., 2011; Milovanovic et al., 2018), and that Syn interaction with SVs depends on its phosphorylation by PKA (Hosaka et al., 1999; Menegon et al., 2006; Cesca et al., 2010). PKA is a powerful regulator of synaptic strength, learning, and memory (Renger et al., 2000; Kandel, 2012; Woolfrey and Dell’Acqua, 2015), and it has multiple molecular targets. Our present study demonstrates that the PKA/Syn pathway selectively promotes the activity-dependent budding of new boutons filled with SVs. This finding drives the hypothesis that dynamic Syn-mediated SV clusters may serve as a presynaptic tag for the activity-dependent formation of new synaptic boutons (Fig. 10*D*).

## Synthesis

Reviewing Editor: Margaret McCarthy, Univ of Maryland School of Medicine Decisions are customarily a result of the Reviewing Editor and the peer reviewers coming together and discussing their recommendations until a consensus is reached. When revisions are invited, a fact-based synthesis statement explaining their decision and outlining what is needed to prepare a revision will be listed below. The following reviewer(s) agreed to reveal their identity: NONE.

Expert reviewers have examined the manuscript and provided a number of useful suggestions that will enhance the impact of the work. The Reviewing Editor agrees with these suggestions and while all of them are no necessary it should be possible to complete a number of them. Please see comments below

The studies address the activity-dependent mechanism of the formation and maturation of new pre-synaptic boutons at the Drosophila NMJ involving PKA/synapsin-mediated synaptic vesicle clustering. The strength of the study is that the group is used multiple approaches to induce neuronal activity in intact larvae, such as elevated temperature or elevated K+ to induce crawling, as well as a temperature-sensitive mutant and dissected larvae preparation. Live imaging of NMJs was performed in anesthetized larvae before and after activity. Live imaging was also followed by immunostaining and electron microscopy to confirm the observations with EM tomography. Overall approach is solid and observations are supported by presented results, suggesting that there are two different pathways that regulate to formation of pre-synaptic immature (filopodia-like without SVs) and mature (rapidly budding filled with SVs) boutons following larvae activity. These mature boutons were also more stable but lacking postsynaptic sites. Finally experiments using PKA modulator forskolin in a combination with synapsin mutant confirmed PKA involvement in synapsin mediated SV clustering in these new boutons. Overall experiments are executed well. However, the impact and interpretation of the results can be substantially improved if the following concerns are addressed.

Concerns:

1. The impact of the study would be higher if it shows that SVs in the newly formed boutons are recycling, which can be done with pH sensitive fluorescent probe. Alternatively, author should remove the speculation on the existence of exo/endocytosis process at these newly formed boutons in the absence of postsynaptic sites.

2. Loss-of-function approach showing that PKA inhibitor would affect the formation of these new boutons with SVs but not ones without SVs would also strengthen the findings.

3. Finally, the analysis of postsynaptic receptor clustering in addition to DLG would be a better indication of assembly of functional postsynaptic sites as DLG can cluster normally in the complete absence of glutamate receptors.

## Author Response

1. The impact of the study would be higher if it shows that SVs in the newly formed boutons are recycling, which can be done with pH sensitive fluorescent probe. Alternatively, author should remove the speculation on the existence of exo/endocytosis process at these newly formed boutons in the absence of postsynaptic sites.

To address this question, we performed additional experiments employing loading of the activity-dependent marker FM1-43. The results of these experiments are presented in new Fig. 7, and they clearly demonstrate that a fraction of newly formed boutons is capable of vesicle recycling.

2. Loss-of-function approach showing that PKA inhibitor would affect the formation of these new boutons with SVs but not ones without SVs would also strengthen the findings.

To address this concern we performed additional experiments employing PKA inhibitor H89. These experiments demonstrated that in fact both types of the boutons are affected by PKA, even though only the new boutons filled with SVs are affected by Syn. These results show that the effect of PKA is more broad that Syn phosphorylation and it involves other targets besides Syn, even though only the growth of SV-filled boutons is regulated via the PKA/Syn - dependent pathway. We have revised the text accordingly.

3. Finally, the analysis of postsynaptic receptor clustering in addition to DLG would be a better indication of assembly of functional postsynaptic sites as DLG can cluster normally in the complete absence of glutamate receptors.

To address this concern, we performed immunolabeling for the postsynaptic receptor GluRIII. We detected the GluRIII puncta at some of the newly formed boutons in the preparations fixed with a 3 hour delay after the stimulation. This new result is presented in the revised figure 1 (new panel 1 F).
